# Immunomodulatory effect of marine lipids on food allergy

**DOI:** 10.3389/fnut.2023.1254681

**Published:** 2023-11-14

**Authors:** Ana G. Abril, Mónica Carrera, Manuel Pazos

**Affiliations:** ^1^Department of Microbiology and Parasitology, Faculty of Pharmacy, University of Santiago de Compostela, Santiago de Compostela, Spain; ^2^Department of Food Technology, Institute of Marine Research (IM-CSIC), Spanish National Research Council (CSIC), Vigo, Spain

**Keywords:** marine lipids, EPA, DHA, immunity, inflammation, microbiota, dysbiosis

## Abstract

Seafood is highly enriched in n-3 long-chain polyunsaturated fatty acids (n-3 LCPUFAs), particularly eicosapentaenoic acid (EPA, 20:5 n-3) and docosahexaenoic acid (DHA, 22:6 n-3), in contrast to the ultra-processed foods included in the modern Western diet that have high levels of n-6 linoleic acid (LA, 18:2 n-6), precursor for the pro-inflammatory n-6 arachidonic acid (ARA, 20:4 n-6). The capacity of marine lipids to reduce plasmatic triglycerides and blood pressure have been well-described. Moreover, recent studies have also raised evidence of a potential regulatory action of marine lipids on inflammation, the immune system, and food allergy (FA). FA is considered one of the main concerns to become life threatening in food safety. The prevalence of this emerging global problem has been increasing during the last two decades, especially in industrialized countries. About a 6-8% of young children and 2-4% of adults is estimated to be affected by FA. The main objective of the current study is to update the existing knowledge, but also the limitations, on the potential impact of marine lipids and their lipid mediators in regulating immunity, inflammation, and ultimately, food allergies. In particular, the focus is on the effect of marine lipids in modulating the key factors that control the sensitization and effector phases of FA, including gut microbiota (GM), inflammation, and immune system response. Results in animal models highlight the positive effect that consuming marine lipids, whether as a supplement or through seafood consumption, may have a relevant role in improving gut dysbiosis and inflammation, and preventing or reducing the severity of FA. However, more systematic studies in humans are needed to optimize such beneficial actions to each particular FA, age, and medical condition to reach an effective clinical application of marine lipids to improve FAs and their outcomes.

## 1 Marine lipids: structure, distribution, and physiological activity

Lipids contained in seafood are characterized by higher proportions of n-3, long-chain (with 20 carbon atoms or more) polyunsaturated fatty acids (n-3 LCPUFAs), particularly eicosapentaenoic (EPA, 20:5 n-3) and docosahexaenoic acids (DHA, 22:6 n-3), in contrast to the lipids found in edible terrestrial animals and plants. Omega-3 polyunsaturated fatty acids (n-3 PUFAs) are a class of fatty acids with more than one double bound, or unsaturation, in which the first double bound is located between the third and the fourth carbon atom, starting from the terminal methyl group. It is well-accepted that EPA and DHA are endogenously biosynthesized at very low rates by humans and fish species (1, 2); therefore, diet constitutes an essential source to incorporate those marine lipids either for humans or fish species.

Traditionally, marine lipids have been incorporated into our diets with the intake of seafood. The content of marine lipids is highly dependent on the fish species, feeding, and season, although in general, the flesh of fatty fish is endowed with a more elevated absolute content of marine lipids (EPA + DHA) compared to that of lean fish, such as cod, hake, and pollock. This fact is due to the capacity of fatty fish, such as salmon, sardine, mackerel, or tuna, to store lipids in the flesh; meanwhile, the liver is the main lipid storage reservoir in lean fish. Thus, as an example, the content of EPA + DHA per 100 g of the flesh ranged 1.79–1.84 g for mackerel, 1.59–2.14 g for salmon, 0.98 g for sardine, levels approximately 4–10 times higher than those found in the flesh of the lean fish species cod (0.15–0.24 g of EPA + DHA), catfish (0.17–0.28 g of EPA + DHA) and haddock (0.15 g of EPA + DHA) (2).

Additionally, the consumption of nutraceutical supplements enriched in fish oil or non-fish sources, such as oil from microalgae and marine invertebrates, specifically copepods and krill, has becoming nowadays very relevant to compensate for possible deficiencies of marine lipids in our diets (2). Microalgae are the prime producers of EPA and DHA in the aquatic habitat and are essential to transfer these fatty acids to the rest of the food chain. However, it should be noted that not all microalgae can produce EPA and DHA, and among those with this capacity, the content of total lipids and EPA/DHA fatty acids may be highly different between species but also within the same species. Several environmental factors highly influence the microalgae lipid metabolism and, consequently, the production of EPA and DHA, such as salinity stress, pH, temperature, light, and the availability of nutrients, vitamins, and hormones (3). The content of total lipids has been reported to represent up to 50-77% in *Schizochytrium* and 30-50% in *Crypthecodinium*, two relevant microalgae in human nutrition, although both species are essentially producers of DHA (up to 97 % of total lipids) rather than EPA (0-2 %) (2). However, new investigations have shown an important improvement in EPA and DHA co-production with *Schizochytrium* by using a specific strain (4), and optimizing nutrients carbon sources (5), so attempting to match EPA/DHA ratios found in fish species. It is relevant to mention that EPA/DHA ratios may have a key relevance in the physiological activity of marine lipids. For instance, marine lipids with different proportions of EPA and DHA have shown differences in the cardiovascular effect (6–8) and the protection against inflammation and oxidative stress (9, 10).

Antarctic krill (*Euphausia superba*) has also received important attention as a source of marine lipids due to its abundant availability, high content in n-3 LCPUFAs (with reported values as elevated as 60% of total fatty acids), and relatively large size compared to copepods and other zooplankton (2).

Additionally, marine lipids from krill are mainly constituted by phospholipids (reaching up to 80% of total lipids), a fact that induces higher bioavailability compared to marine lipids derived from fish, mainly composed of triglycerides.

An important number of the physiological effects of marine lipids are induced by the integration of EPA and DHA into the cell membranes. The incorporation of marine lipids into the phospholipid membranes of the cell provides a specific environment that modulates physical properties, such as fluidity, and the function of membrane proteins like transporters, signaling enzymes, and receptors, but also induces that EPA and DHA can be used as substrates for the generation of effective lipid mediators with the capacity to modulate the immune system and resolve inflammation (i.e., eicosanoids, resolvins…) (1). Marine lipids can also alter membranes of different cells, including immune cells, neurons, hepatocytes, adipocytes, and cancer cells, so affecting intracellular signaling pathways and cell response. On the other hand, other investigations have shown that EPA and DHA can directly interact with specific G-protein coupled receptors, such as GPR120, which is extremely high present in inflammatory macrophages and on adipocytes, and whose interaction with marine lipids can induce effective anti-inflammatory and insulin sensitizer effects (11).

Regarding their physiological activity, clinical evidence supports that EPA and DHA have an effect in lowering the risk of cardiovascular disease, particularly coronary heart disease. Increasing evidence from human and animal investigations also suggests that marine lipids may have an effect in reducing risk of developing some cancers, particularly breast and colorectal, and protecting against different inflammatory conditions and the development of childhood allergic diseases (1, 12). The following sections will be focused on the description of the effect of marine lipids and their lipid mediators in the immune system, inflammation, gut dysbiosis, and other factors with potential impact on the development of food allergy (FA).

## 2 Marine lipid mediators, immunity, and inflammation

The two principal PUFA families, n-6 and n-3 series, are the main substrates for the formation of the bioactive lipid metabolites oxylipins. Based on the number of carbon atoms, oxylipins can be grouped in octadecanoids, metabolites derived from 18-carbon PUFAs; eicosanoids, metabolites derived from 20-carbon PUFAs; and docosanoids, metabolites derived from 22-carbon PUFAs. Oxylipins are synthesized during normal cell regulation, but even more importantly, after cell activation in pathological conditions such as stress, allergy, fever, and inflammation, functioning as signaling molecules for the control of different processes implicated in cell metabolism and immune response. Some of the biological processes regulated by oxylipins include inflammation, blood coagulation, pain response, apoptosis, cell growth, and blood vessel permeability (13).

**Figure 1:**
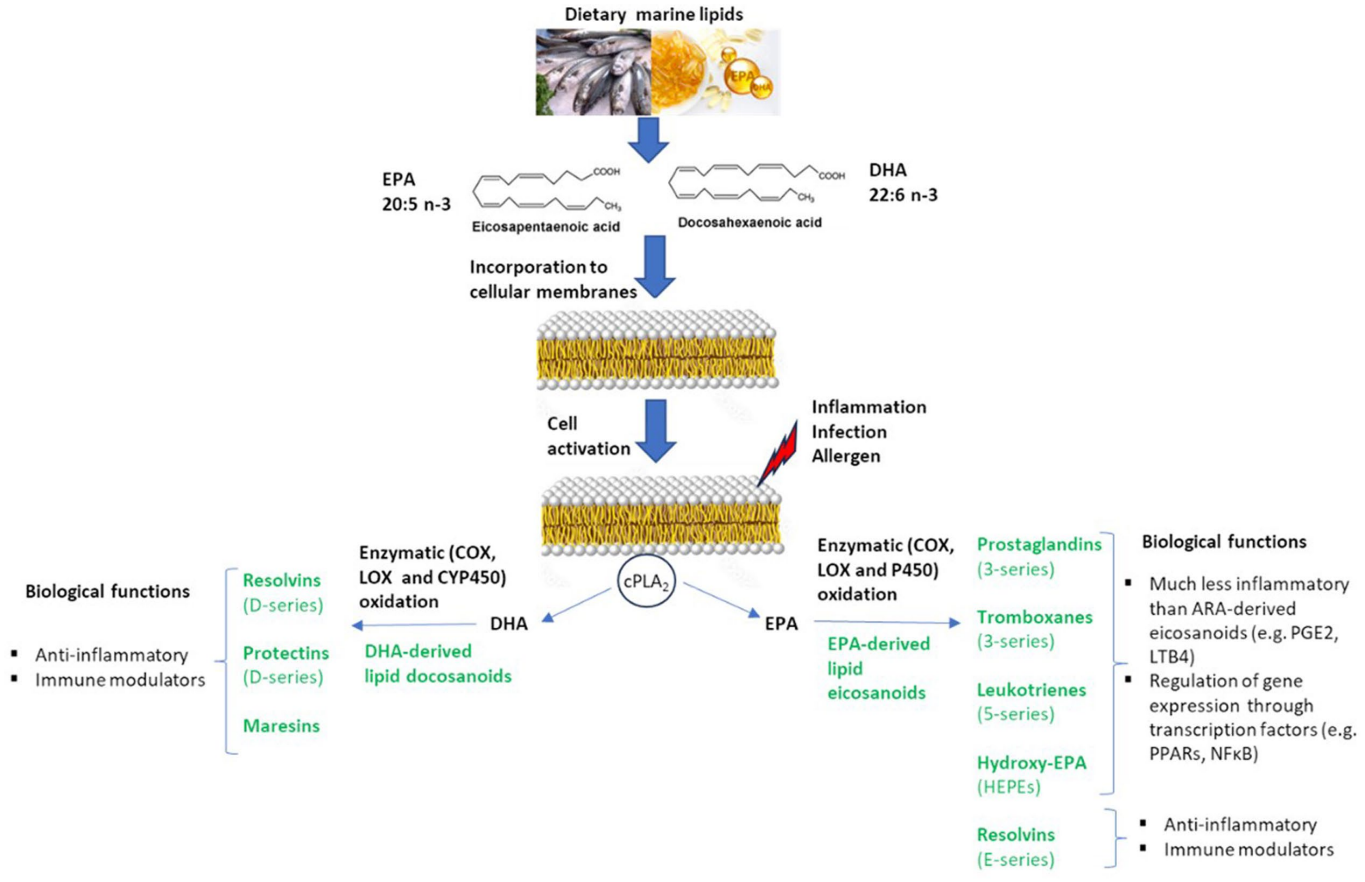
Biosynthesis of marine lipid-derived bioactive lipid mediators with biological capacity to modulate inflammation, immunity and food allergy.

Figure 1 summarizes the metabolization of dietary marine lipids into bioactive oxylipins relevant for the regulation of inflammation, immunity, and allergy. Oxylipin formation, including those EPA-and DHA-derived, is cell activated by the activity of a phospholipase, generally the cytosolic phospholipase A2 (cPLA2), that releases fatty acids from the phospholipid bilayer of cell membranes, which constitute the main pool of bioavailable precursors of oxylipins (13). Then, the free PUFAs are metabolized into oxylipins via non-enzymatic or enzymatic oxidation processes. Cyclooxygenase (COX), lipoxygenase (LOX), and cytochrome P450 (CYP450) epoxygenase enzymes are the main catalyzers for the production of bioactive lipid mediators via enzymatic pathways (14). It is well-recognized a competition between n-6 and n-3 PUFAs in the production of oxylipins, so the incorporation of EPA and DHA into the phospholipid membranes induces a higher generation of lipid mediators derived from EPA and DHA-, meanwhile lowering the production of n-6 arachidonic acid (ARA)-derived eicosanoids, which are generally categorized as proinflammatory mediators (15).

Prostaglandins (PGs) from 3-series, thromboxanes (TXs) from 3-series, leukotrienes (LTs) from 5-series, resolvins (Rvs) from E-series, and hydroxy-EPAs (HEPEs) are principal EPA-derived eicosanoids; meanwhile, resolvins (Rvs) from D-series, protectins (PDs) and maresins (MaRs) are main DHA-derived bioactive oxylipins (Figure 1). The eicosanoids derived from EPA and DHA are considered to be less inflammatory compared to eicosanoids from ARA, or even with anti-inflammatory properties. Thus, the marine lipid-derived mediators Rvs, PDs, and MaRs have shown capacity as lipid mediators in the resolution of inflammation and the regulation of immunity, either *in vitro* or *in vivo* (14). Marine lipids, and their lipid mediators, also exert anti-inflammatory actions via regulation of inflammatory gene expression through their effects on transcription factors such as peroxisome proliferator-activated receptors (PPARs) and nuclear factor kappa B [NFκB (14, 16)].

The principal eicosanoids from ARA are the PGs and TXs from 2-series generated by COX pathways; LTs from 4-series, hydroxyeicosatetraenoic acids (HETEs) and lipoxins generated by LOX pathways; and HETEs and epoxyeicosatrienic (EETs) acids generated by cytochrome P450 epoxygenase pathways (14, 17). The incidence of inflammatory episodes is associated with excessive production of the n-6 ARA-derived leukotriene B4 (LTB4) and prostaglandin E2 (PGE2) (15). In the modern Western diet, essentially enriched in ultra-processed foods, high levels of ARA can be maintained in cellular membranes due to its low content of n-3 PUFAs and high levels of the n-6 linoleic acid (LA, C18:2n-6), that is the main precursor for n-6 ARA (15, 17). On the contrary, dietary marine lipids have shown an effect on modulating the profile of oxylipins and decreasing the levels of proinflammatory cytokines. Previous investigations in animal models demonstrate that the consumption of marine lipids causes a replacement of the n-6 ARA by the n-3 EPA and DHA in membranes from erythrocytes and tissues, and that accordingly increased the content of EPA-and DHA-derived oxylipins, while reducing the production of n-6 arachidonic-derived eicosanoids (9, 18, 19). This modification of the profile of oxylipins toward the production of EPA-and DHA-derived mediators, instead of the generation of the pro-inflammatory ARA-derived eicosanoids, agreed with an improvement for parameters of inflammation, oxidative stress, and protein oxidation (9, 10). The consumption of marine lipids also increased the content of EPA and DHA in inflammatory cell membranes and subsequently reduced the generation of ARA-derived eicosanoids that induce the production of proinflammatory cytokines in macrophages and cause pain and vasodilation (15).

In allergic diseases, ARA-derived eicosanoids are generated from cells during allergic responses and clinical symptoms, including bronchial spasms, blood pressure variations, and diarrhea (20). In the immune system, eicosanoids are produced predominately by antigen-presenting cells of the immune system and have specific effects on dendritic cells. EPA-and DHA-derived lipid mediators have shown pro-resolving functions by inhibiting neutrophil accumulation into inflammatory sites and promoters of apoptotic cell clearance by macrophages (20). These mediators can regulate many types of inflammatory and immune cells, including T cells, dendritic cells, eosinophils, and mast cells. Particularly, the EPA-derived resolvin E1 (RvE1) and DHA-derived protectin D1 (PD1) promote phagocyte removal during acute inflammation by regulating leukocyte infiltration, increasing macrophage phagocytosis of apoptotic polymorphonuclear neutrophils, and enhancing the appearance of phagocytes carrying zymosan in lymph nodes and spleen (21). DHA-derived maresins (MaR) are macrophage mediators in resolving inflammation, being MaR1 identified first in self-resolving inflammatory exudates with human macrophages via 12-LOX-initiated mechanisms (22). This investigation suggested the beneficial actions of maresins in tissue homeostasis, inflammation resolution, wound healing, and host defense. Dyall et al. (17) have recently published a study about PUFA-related lipid mediators, including those derived from the EPA and DHA, that provides updated information about their biosynthesis, structures, and biological function.

Several studies have shown that n-3 marine PUFAs play a vital role in reducing the risk of infants developing FA when pregnant and lactating women are supplemented with fish oil (23). Accordingly, Kunisawa et al. (24) reported that n-3 fatty acids play a role in alleviating FA by converting EPA into 17,18-epoxyeicosa-pentaenoic acid, in the gut of a murine FA model. This EPA-derived metabolite showed an *in vivo* anti-allergic effect by decreasing the incidence of allergic diarrhea due to impairment of mast cell degranulation without affecting allergen-specific serum IgE. In the next sections, factors and mechanisms responsible for the immune response in FA, as well as the potential role of marine lipids to mitigate hypersensitivity to food allergens, will be deeply described.

## 3 Food allergy

FA is a relevant and communal health concern according to the World Health Organization (WHO). In the European Union (EU), the European Food Safety Authority (EFSA) has indeed identified 14 foods as major allergens: wheat, fish, mollusks, crustaceans, milk, eggs, peanut, nuts, soybean, sesame, mustard, celery, lupin, and sulfur dioxide/sulfites. Up to the present time, the unique demonstrated and successful remedy for this class of sensitivity is to follow a regimen restricted in the allergenic food and the products. The clinical symptoms of FA emerge within 60 min of consumption and involve nausea, vomiting, acute urticaria, diarrhea, asthma, and wheezing. In the gravest situations, potentially life-threatening anaphylactic shock can occur (hypotension, trouble breathing, and weak pulse) (25, 26).

### 3.1 The allergic immune response: sensitization and effector phases

**Figure 2:**
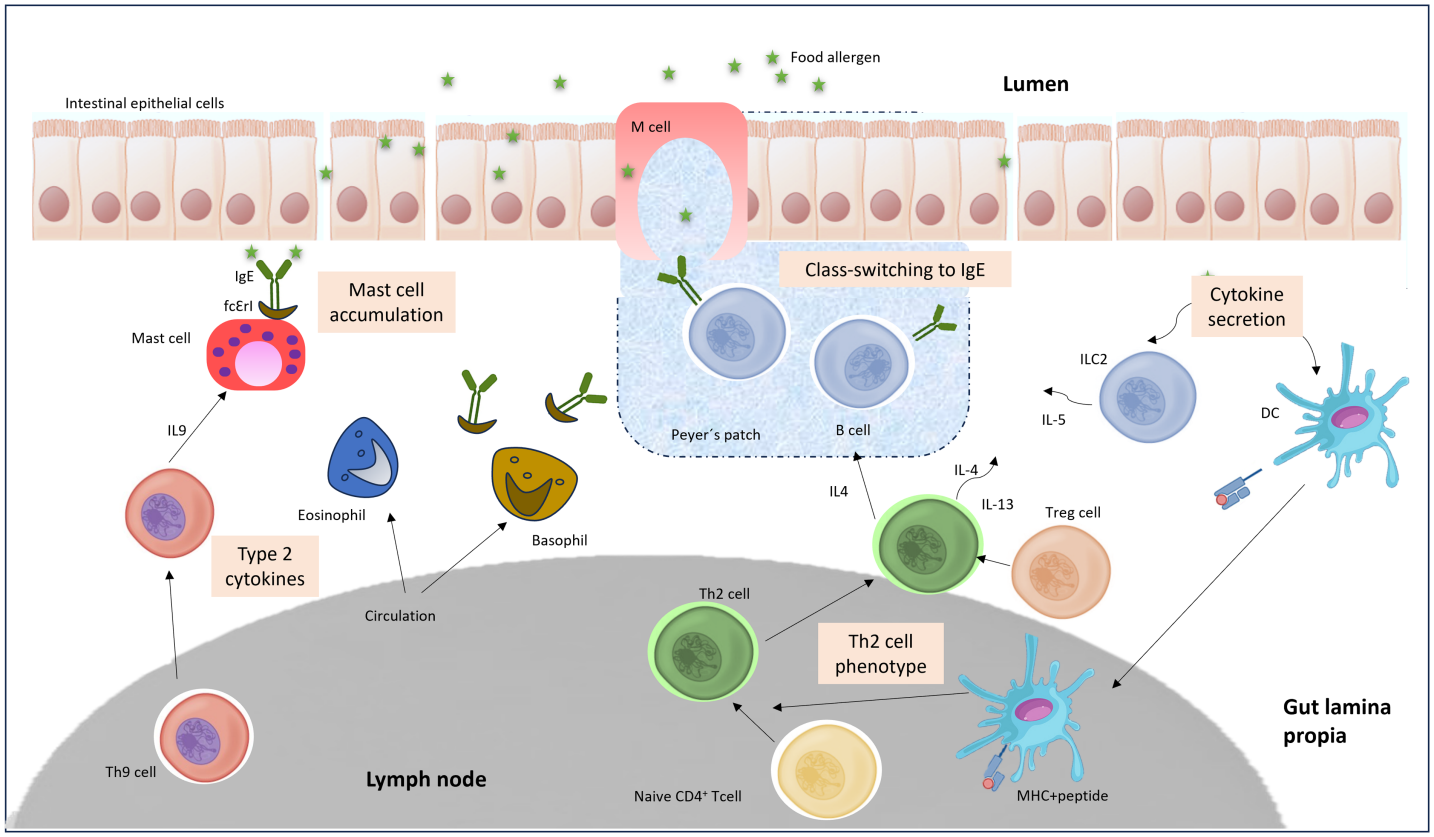
TH2 cell-mediated inflammatory response to oral antigen in the gut.

FAs are attributable to a distorted reaction of oral tolerance to food antigens. Early works on allergic sensitization indicated that this process is initiated because of the alteration of the regular oral tolerance in the gastrointestinal (GI) tract by the regulatory T cells (Tregs), that generate the preservation of tolerance to foodstuff antigens, which are distorted and substituted by the initiation of an effector immunological reaction diverged toward an allergenic T cell reaction headed by Th2 cells. Then, the effector Th2 cells are proposed to control the IgE response through the secretion of IL4, IL5, and IL13 cytokines. These specific cytokines are essential for B cell class converting, production of specific IgE, extension of allergic effector cells, and occurrence of clinical manifestations (27). Figure 2 shows a summary of the main factors and triggers that induces a FA immune response.

However, current progress in high-throughput methodologies and data analysis is moving this simplistic vision to a more complex model in which different T-cell lineages, such as T-cell receptor (TCR)αβ, TCRγδ, regulatory T cells (Tregs), and follicular T helper cells (Tfhs) are involved (28). Interestingly, the subset Tfh13 has also been demonstrated to be required to produce high-affinity IgE, which is responsible for the anaphylactic reactions (29).

Concerning the possible origin of tolerance rupture, external damages and innate triggers have been indicated as in charge of the cytokine liberation by intestinal epithelial cells (IECs) of thymic stromal lymphopoietin, IL-33 and IL-25, that perform a crucial task in the initiation of allergic reactions at the mucosa line (30). The combined emission by IEC has been exposed critical for the sensitization to food antigens, while the liberation of some of these cytokines can continue a determined FA (31). Additionally, IECs have described as a main resource of eotaxin in the intestine that controls the release of eosinophils, which are correlated to the gravity of the intestinal symptoms against food allergens (32).

The immune mechanisms of FA maintenance continue to be unclear. In this concern, the main task executed by the allergen-Th2 cells in starting the allergic reply has newly been defined (33). The authors found a subgroup of allergen-specific memory Th2 cells distinguished by a specific signature (CD45RB-CD27-) and the secretion of CD161, CD49d, CRTH2. Such cells present several operational characteristics different from conservative Th2 cells, involving the mutual liberation of different Th2-associated cytokines (IL13, IL4, IL9, IL5). Additionally, this affected T cell subgroup has demonstrated an established allergic-associated phenotype.

Animal studies demonstrated that the ingestion of antigens via oral treatment is operative in the generation of oral tolerance of food. Therefore, mice consuming oral proteins prompts peripheral allergen-mediated Foxp3+ CD25 + - Tregs and provokes modulatory reactions dependent on TGF-b secretion (34). More Treg subgroups that have been related to the stimulation of oral tolerance are Th3 and Tregs type 1 (Tr1). The Tr1 cells are categorized by exhibition of the CD49b and the LAG-3 in the expression of absent CD25 and Foxp3 manifestation (35). Then, Th3 cells are recognized by the superficial production of latent-related peptides, and they besides have restrictive actions due to TGF-b production (36). In both cases, their responsibility in oral tolerance stimulation has been associated with the defeat of immunological reactions via IL-10 liberation (37). While Tregs specificity appears to be tolerance contrasted with allergy to aero-specific Tregs to foodstuff antigens have not ocurred exposed changed in allergic patients contrasted to healthful persons (38). These details limit the current comprehension of the function of Tregs in FA in persons.

Furthermore, accumulative confirmation proposes that sensitization in FA can be established by non-oral ways, such as through the skin. Thus, primary cutaneous contact with food antigens across a disturbed skin wall endorses allergic sensitization before the primary digestion of food, as divergent to the tolerogenic character of oral contact. Thus, double interaction indicates that contact with food allergens across disturbed skin endorses sensitization, while primary experience with food allergens across oral via stimulates tolerance (39). In effect, there is a robust connection between sensitization to food allergens and eczematous skin or atopic dermatitis (40). However, while topical contact has been suggested as a way of sensitization in FA, investigational data have established that skin is not integrally sensitizing, as skin presentation of food allergens in the deficiency of peripheral adjuvants (41). Additionally, cutaneous sensitization to food allergens may necessitate the influence of supplementary elements, such as skin wall alterations (42) and the incidence of adjuvants like toxins created by microorganisms growing in the skin (43). These confirmations sustenance the premise that, in several circumstances of skin wall inflammation or alteration, food allergens sensitization can be provoked by the skin.

Regarding the other T-cell subsets (TCR)αβ, TCRγδ, and follicular helper other T (Tfh) cells studies from food-allergic patients and murine models indicate that Th2 and some Tfh cells induce the production of IgE by B cells. γδ T cells in healthy and allergic subjects harbor both IgE-enhancing and suppressive subsets, positioning them as master regulators of oral tolerance (44–46). Recently, Merino et al. (47) have achieved the allergic sensitization of BALB/c mice with a systemic specific IgE and a perturbed TCRγ chain repertoire in Peyer’s patches using supplementation with the major fish allergen beta-parvalbumin and alum as an adjuvant. Preliminary mRNA massive sequencing experiments have linked the sensitization to an increase in TCR-Vγ1 expression in Peyer’s patches of the sensitized mice. The involvement of these TCR-Vγ1+ cells in asthma and airway inflammation has also been reported (44). All these data suggest a role of this T-cell subset in allergic IgE-mediated response. However, the molecular mechanisms underlying the control of IgE-mediated immune response by γδ T-cells and the role of each γδ T-cell subset in allergic disease remain elusive. It must be stressed that opposite to the FA studies in humans limiting the study to immune cells in peripheral blood, the use of mouse models allows to target the mucosal and compare it with the systemic level (48).

Besides the dual assumption, the hygiene hypothesis furthermore increases attention. Industrialization and urbanization, together with an intensification of primary contact with cleaning products and antimicrobials, increase exposure to chemical diversity, reduce microbial outdoor exposure, and alter human microbiota (49). Primary microbial contact has repercussions with FA, as confirmed with germ-free mice, because these animals are extremely predisposed to anaphylactic reactions to food. The contact of neonatal babies with the maternal vaginal area, the outer bacteria and breast milk, produces the induction of microbial receptors and the expansion in the mucosa of tolerogenic immunological nets that defend of allergic responses. Augmented ratios in birth antibiotic use and cesarean deliveries have been decreasing the neonate microbial contact to the maternal microbiota and the soil pets’ microbiotas and farming animals’. Such as circumstances have been related to human health with the amplified transition from infectious to non-communicable illnesses, including FAs. Moreover, nutrition is the major powerful modulator of microbiota, prompting variations in microbiome and bacteria-resulting metabolites. Truthfully, short-chain fatty acids (SCFAs) and dietary fiber endorse regulatory immune reactions and protein breaks by the intestinal microbiome give rise to amino acid-resulting metabolites with immunological modulatory features (50). All these aspects are discussed in detail in the next section.

### 3.2 Food allergy and gut dysbiosis

It is currently well-established that individuals suffering from FAs display an intestinal microbiota with a different microbial composition than in healthy conditions. The GI tract is composed of thousands of microorganism species, including bacteria, protists, fungi, and viruses as bacteriophages, each one with a specific direct or indirect role in the prevention of the development of allergies. The gut microbiome is modified from birth to death, and many factors are involved in these changes. Factors associated with GI microbial composition modifications that are involved in the development of allergies are the diet, the manner of birth, environmental interactions and pet exposition, and administration of medical therapies such as the use of antibiotics (51). Strict maintenance of microbiota composition is required to avoid food intolerances as the alterations can lead to dysbiosis, consequently producing inflammation and pathogenesis in the gut resulting in the development of FA (52). Dysbiosis in the gut produces GI tract malfunction resulting in occasions in a breach in the intestinal barrier, modifying GI permeability. This can cause that gut antigen may reach the bloodstream producing allergic reactions in other organs (53). The modulation of gut microbiota (GM) seems to be a good strategy for treating allergies (54, 55). Different studies of microbial transplants in mice and humans have demonstrated the implication of dysbiosis in FAs. Fecal transplants and other bacterial therapeutic approaches such as probiotics, prebiotics, and symbiotics can provide prevention and treatment of FAs (52).

Those factors affecting intestinal microbiota are involved in microbiome development during life. In healthy conditions, *Bifidobacterium* and *Lactobacillus* genera predominate in the post-natal gut microbiome, which provides a healthy immune regulatory response involved in gut T effectors (helper T cells) and secretion of IgAs. With the introduction of solid food in infants, appear new species of the orders Clostridiales and Bacteroidetes, which play a role in the suppression of IgE typically found in FA (56). The diminution of Clostridiales, and more specifically, species of the genera *Leuconostoc*, *Weissella,* and *Veillonella* during the first year of life, make babies can suffer FA (51, 56–58). Moreover, different bacteria, such as Enterobacteriaceae and Parabacteroides, and families, such as Lachnospiraceae and Ruminococcaceae, can improve these FA conditions (59). Lacto-dysbiosis also influence the development of FA with the reduction of lactate-utilizing bacteria, such as *Eubacterium* and *Anaerostipes*, that produce butyrate (57).

Several studies have evaluated the association between GM and the increase of allergies. It was demonstrated that species of *Clostridiales* and *Bacteroidales* taxa inhibit in mice the development of FAs via the induction of the retinoic orphan receptor gamma T (ROR*γ*t^+^) in T regulatory cells (Tregs) decreasing GATA-31 (GATA binding protein 31) Tregs and IgE while enhancing IgA production (56, 60). Moreover, as mentioned above, fecal microbial transplantation of beneficial commensal bacteria can be used to prevent and treat allergies by inducing RORγt+, promoting tolerance to dietary antigens (57, 58, 61).

Species of *Lachnospiracea*e, *Streptococcaceae,* and *Leuconostocaceae* are abundant in the GI of children with egg allergies. It was studied how having older siblings or a pet dog reduced the possibility of developing egg allergies in infants (62). Another study evaluated the importance of acquiring commensal microorganisms from mothers during the process of birth by vagina delivery, while cesarean delivery leads to the development of FAs in children (51).

A study was performed on 18 pairs of twins from 6 months to 58 years of age. In 13 of these pairs of twins, one of them suffered from a FA, and in the remaining 5 pairs, both twins suffered from FA. Results showed a higher number of differences in bacterial composition in fecal samples between the first set of twins with and without FA individuals. This research has demonstrated the protective effect of some microbial families preventing the development of FA and their role in the GI (59). Moreover, there were found higher concentrations of specific commensal-produced metabolites, such as diacylglycerol, in healthy twins. The main responsible for diacylglycerol production in GI was *Phascolarctobacterium faecium,* while *Ruminococcus bromii* plays a role in both starch digestion and the metabolism of amino acids and sterol (59).

Studies in germ-free (GF) mice have also shown the importance of microbial GM in the prevention and development of FAs (63). GF mice received fecal microbiota transplants from animals with FAs and developed the same type of FA as the donor. Other analyses were performed to study the influence of *Anaerostipes caccae* in GF mice. The animals were protected from the development of FA. In addition, orally administered *Citrobacter koseri* increased systemic allergic symptoms in allergic mice by inducing IL-33 release (64). Furthermore, a murine model (Il4raF^709^; C.129×1-Il4ratm3.1Tch) that is prone to FA and harbors a gain-of-function mutation in the Interleukin-4 receptor-alpha chain (IL-4Ra) is susceptible to FA associated with changes in the GM (65). This mouse model was the tool of several studies to evaluate the relationship between FA, microbiome composition, and the corresponding immune responses produced (57, 58, 66).

## 4 Marine lipids, gut dysbiosis, and food allergy

### 4.1 Marine n-3 PUFAs influence on gut microbiota

GM dysbiosis is directly related to the development of inflammatory disorders such as allergies. At this point, the consumption of specific food supplements may modulate the GM affecting human health. In this sense, the marine environment is an important source of bioactive compounds, including polysaccharides such as algae-compounds (alginate, fucoidan) and animal-derived polysaccharides (chitin, chitosan) active peptides and PUFAs (67).

Dietary lipids display different results on GM dysbiosis depending on the type of fatty acid (68–70). Some microorganisms use n-3 PUFAs to produce secondary metabolites. Changes in GM have been observed after diet supplementation with marine lipids. Lactic acid-producing bacteria, among others, can produce PUFA-derived intermediate metabolites, and these PUFA-derived bacterial molecules provide beneficial anti-obesity and anti-inflammatory effects (71). Particularly, marine n-3 EPA and DHA have an important role in the modulation of the diversity and abundance of GM (72–74), and such impact on GM has been related to an attenuation of the metabolic dysfunction associated with obesity when supplemented with marine lipids (75). Moreover, marine lipids improve the barrier function of the intestinal mucosa and intestinal microenvironment, and also increase intestinal mucosal thickness. In addition, marine lipids are involved in weight loss by modulating fat metabolism genes (76). The metabolism and absorption of n-3 PUFAs are influenced by GM; otherwise, the information about the direct implication of these lipids in GM is limited. The composition and diversity of GM is modulated by marine lipids, modifying the present proinflammatory mediators and balancing the levels of SCFAs (77).

Studies using animal models (Table 1) have demonstrated a clear association between dietary fatty acids and changes in GM (97). Dietary intake of fish oil can significantly affect the diversity of the intestinal flora compared to sunflower oil. This effect is due to the high levels of n-3 PUFAs present in fish oil, particularly EPA and DHA, which can modify the GM (98, 99). In addition, n-3 PUFA intake increases the growth of Bifidobacteria while decreasing the growth of Enterobacteria, providing the inhibition of inflammatory mediators linked to metabolic endotoxemia (100). Bifidobacteria and Lactobacilli, among other anaerobic bacteria, partially metabolize n-3 PUFAs in the distal intestine. This metabolism can affect the distribution of the intestinal flora, as it promotes the growth of beneficial bacteria, such as *Bifidobacterium*, while inhibiting the growth of potentially harmful bacteria (101, 102). It has been elucidated how marine lipids increase the richness of Bifidobacteria in the gut of male rats (103) and can prevent GM dysregulation in mice (104), increasing the abundance of commensal bacteria that produce lactic acid and Bifidobacteria in the gut of the mice fed a high-fat diet (84, 105).

**Table 1:** Summarized studies that evaluate the influence of marine lipids on gut microbiome (GM), immunity and inflammation [Modified from Costantini et al. (78) and Wang et al. (67)].

Study	Diet	Sampling	Outcomes on GM	Outcomes on immunity and inflammation	Ref.
Mice	Different fish oil doses (10 mg/kg or 5 mg/kg) for 2 weeks	Imprinting Control Region mice	Fish oil supplements decrease the abundance of Firmicutes phylum		Yu et al. (72)
Mice	High-fat (HF) diet (45%) for fish oil or lard	C57Bl/6 Wild-type germ-free mice	Fish-oil supplementation increases the abundance of Lactobacillus genus and *Akkermansia muciniphila* species. However, HF diet increases abundance of *Bilophila* genus.	Mice fed a HF diet have increased TLR activation in the systemic circulation, increased white adipose tissue inflammation, and impaired insulin sensitivity compared to mice fed fish oil.	Caesar et al. (79)
Mice	Corn oil diet or corn oil + fish oil diet for 5 weeks	C57BL/6 mice	n-6 PUFAs increase Enterobacteriaceae family, and n-3 PUFA increase *Lactobacillus* and *Bifidobacteria* genera abundance.	n-3 PUFA, reversed intestinal damage and inflammation during infection-induced colitis; however, mice fed n-3 PUFA diet increased infection-induced mortality associated with sepsis	Ghosh et al. (80)
Mice	Fish, soybean, and lard oils for 30 days	Four-week-old male Wistar/ST rats	Fish oil diet increases bile acids in feces and increases the relative abundance of Firmicutes.	Lard diet decreased the expression level of liver ATP-binding cassette subfamily G genes (Abcg5 and Abcg8 genes).	Hosomi et al. (81)
Mice	Fish oil and soybean oil (1.6 fish oil/100 g of diet)	3-months male mice with accelerated senescence (SAMP8)	Fish oil diet decreases the ratio of *E. coli* and *Escherichia*, inhibiting inflammatory bowel diseases.	Fish oil-induced a reduction in serum ALT, AST and liver total cholesterol (TC).	Yamamoto et al. (82)
Mice	Different doses of krill oil (100, 200, 600 mg/ kg/day)	Hyperlipidemic mice	Krill oil diet increases the relative abundance of Bacteroidales and Lactobacillales		Lu et al. (83)
Mice	Diet supplemented with n-3 from microalgae (1 g EPA + DHA/100 g)	C57BL/6 J mice	n-3 PUFA diet increases the richness of *Bifidobacterium* and *Lactobacillus* genera, increase cognition, and dampens HPA axis activity.	n-3 PUFA did not affect TNFα and IL-10 levels in unstimulated splenocytes. After a splenic stimulation with LPS, revealed a significant effect of diet on the release of TNFα while there was no significant effect of diet on IL-10 release.	Robertson et al. (84)
Mice	HF diets supplemented with Menhaden fish oil (60%/5.3% kcal fat).	Male C57BL/6 mice	Fish oil increases Bacteroidetes, enhancing the intestinal barrier functions and related obesity dysfunctions.	Beneficial effect of fish oil in intestinal-derived inflammatory LPS signaling (via decreased TLR4 expression) and decreased adipose tissue hypoxia (via decreased HIF1α expression), EAT mRNA expression and/or serum concentrations of cytokines (i.e., leptin and resistin)	Monk et al. (75)
Mice	HF diets were supplemented with fish/ krill oil (10/40% kcal fat 12 weeks)	10-week-old male ICR mice	Supplements decrease weight gain, liver index, and total cholesterol in HF mice and decrease the ratio of Firmicutes to Bacteroidetes.	HF diet-fed mice: significant increases LDL-C, TC and TG, a decrease in HDL-C compared with the control group.	Cui et al. (85)
Mice	PUFA from *Spirulina platensis* (150 mg/ kg/day) 4/8 weeks	HF diet-induced hyperlipidaemic rats	Increase of *Turicibacter, Clostridium*_XIVa, and *Romboutsia*, which were positively associated with lipid metabolism.	SPL55 (Spirulina platensis 55% ethanol extract)treatment stabilized serum HDL-c levels. SPL55 improved the expression levels of lipid genes by up-regulating AMPK-α and down-regulating SREBP-1c and HMG-CoA	Tong et al. (86)
Mice	Fish oil (2.5 mL/kg)	Four-week-old male WT and Fmr1 KO mice	Autistic behaviors in Fragile X syndrome were improved with fish oil administration.		Guo et al. (87)
Mice	Soybean and fish oils (18% EPA and 12% DHA) for 10-weeks.	Twenty-four Wistar rats, aged 6 weeks and weighing 300–350 g under chronic sleep deprivation	Fish oil ameliorates the effects of gut dysbiosis in neuropsychiatric behaviors.	Fish oil reduced proinflammatory in serum TNF-α, IL-1β, and LPS	Lai et al. (88)
Mice	2% fish oil and 2% soybean oil, and 2% olive oil and 2% soybean oil. Fish oil contained 20.5% (w/w) EPA and 11.2% DHA.	Male Sprague–Dawley rats under chronic mild stress.	Amelioration of depressive-like behavior and GM dysbiosis; increase in the abundance of *Akkermansia*.	n-3 PUFA was not associated with corticosterone level	Tung et al. (89)
Mice	Fluoxetine and fish oil supplementations	Maternally separated male and female Sprague–Dawley rats	Fish oil administration increases Bacteroidetes and Prevotellaceae abundance, while reducing the concentration of butyrate.	Fish oil supplementation decrease plasma corticosterone	Egerton et al. (90)
Mice	Fish oil and algae oil were supplemented (600 mg/ kg/day) for 12 weeks	Ninety-six 10-week-old ICR male mice with an average body weight of 24.1 ± 2.6 g	Marine oil supplementation increases the learning and cognitive abilities of mice.		Zhang et al. (91)
Human	Diabetes diet supplemented with 100 g of sardines 5 days a week for 6 months (n = 17) (~3 g of EPA + DHA)	32 patients diagnosed with type-2 diabetes	Marine lipids decrease the Firmicutes/Bacteroidetes ratio and increase *Prevotella.*	Marine oil administration decreased TNFα levels but did not affect IL-10	Balfego et al. (92)
Human	Daily supplementation of 600 mg of n-3 PUFAs by fish protein diet for 2 weeks	One healthy 45-year-old man	Daily supplementations increase Firmicutes and reduce Bacteroidetes and Actinobacteria phyla. Decrease Faecalibacterium genus and increase *Blautia, Roseburia, Coprococcus, Ruminococcus* and *Subdoligranulum.*		Noriega et al. (93)
Human	Cow’s milk or infant formula w/o 5 mL/day of fish oil until the 12th month	One hundred and fourteen 9-month-old infants	Fish oil in cow’s milk displays differences compared with no supplementations. Differences were not found in infant formula groups.		Nielsen et al. (94)
Human	Daily supplementation of 5 mL fish oil (1.6 g EPA + DHA) or sunflower oil (3.1 g linolenic acid, n-6) for 9 months	One hundred and thirty-two 9-month-old infants	Fish oil significantly modifies GM in comparison with sunflower oil. However, only affects children who had stopped breast-feeding before the study.		Andersen et al. (95)
Human	Administration of nutritional with a fish and safflower blend oil for 10 weeks	32 premature infants with enterostomy	Decrease of pathogenic bacteria as *Streptococcus, Clostridium, Escherichia, Pantoea, Serratia*, and *Citrobacter* genera.		Younge et al. (96)

Most of the research on marine lipids in microbiota focuses on Bacteroidetes and Firmicutes phyla in animal models as they are the two principal phyla of bacteria in the human gut microbiota (GM). n-3 PUFAs from fish oil decrease the population of Firmicutes (72). Moreover, an increase in the Firmicutes/Bacteroidetes ratio (F/B ratio) is related to metabolic conditions such as obesity, insulin resistance, and non-alcoholic fatty liver disease because of the synthesis of SCFAs. Marine lipids exert a positive effect by increasing the production of anti-inflammatory compounds, such as SCFAs (106). Butyric acid-producing bacteria degrade nonfermentable dietary fibers into SCFAs, such as butyrate (107). The addition of marine lipids to Salmonella-infected mice substantially rises the production of SCFAs by increasing the abundance of SCFA (butyrate)-producing genera (108, 109), such as *Blautia, Bacterioides, Roseburia,* and *Coprococcus* (110) and *Lactobacillus* in the mouse intestinal tract (111). In addition, it has been studied how an imbalanced n-3/n-6 PUFA intake provides dysbiosis (112, 113). n-3 PUFAs modify the content of commensal gut bacteria, particularly *Akkermansia* (112). Caesar et al. (79) demonstrated that the type of dietary fat can significantly impact the GM. The study found that rats fed a fish oil diet had a higher abundance of *Lactobacillus* and *Akkermansia muciniphila*, while those fed a lard diet had higher levels of *Bilophila*. These findings suggest that fish oil may have anti-inflammatory effects through its impact on the GM. These results are consistent with other studies showing that marine lipids can modulate GM and improve metabolic health (79).

Ghosh et al. (80) investigated the effects of dietary fat on GM in mice. The researchers found that diets supplemented with fish oil decreased Enterobacteriaceae and Clostridia abundance. In addition, Monk et al. oil (75) evaluated the consequences of the administration of fish oil in GM content and their effects on the epithelial barrier. Male mice were daily fed a high-fat diet based on meat or a high-fat diet based on fish oil. The supplementation with fish oil provided an increase in the content of Firmicutes and Bacteroidetes phylum. Moreover, oral glucose tolerance was improved, providing intestinal health. Furthermore, n-3 PUFAs can improve symptoms of Inflammatory bowel disease (IBD) by reverting the microbiota composition, increasing the abundance of the *Escherichia*, *Faecalibacterium, Streptococcus, Sutterella*, and *Veillonella* genera (114) and inhibiting the growth of the *Bacteroides, Flavobacterium*, and *Oscillospira* genera resulting in the decrease of Firmicutes/Bacteroidetes (F/B) ratio (115). Moreover, Yamamoto and colleagues (82) have evaluated the effect of fish oil administration on aging. It is known that intestinal inflammation is affected by aging; otherwise, it was decreased by a moderate-fat diet supplemented with fish oil in mice. In addition, it was shown a decrease in the bacteria that are involved in energy consumption with aging. Other studies evaluated different fat sources, such as soybean oil, lard, menhaden oil, or tuna oil (81). The results showed that dietary fats affected differently the relative composition of fecal microbiota and bile acid metabolism, and in particular, menhaden oil increased fecal bile acids excretion compared with soybean oil and lard diets. Fecal BA excretion was found to be directly associated with the relative abundance of Firmicutes, and negatively associated with the relative abundance Bacteroidetes. This investigation also suggested that the impact of fish oils on the fecal microbiota may vary greatly correlated to the ratio of EPA to DHA and the composition of fatty acids other than n-3 PUFA. Guo and colleagues (87) administrated fish oil to four-week-old male WT and Fmr1 KO mice and demonstrated that fish oil ameliorates autistic behaviors and gut dysbiosis in fragile X protein (FMRP)-deficient mice (87). Moreover, dietary marine lipids have also been involved in inhibiting the production of proinflammatory mediators and in the restoration of GM, which is produced by life stress (90). Tung and coworkers (89) have studied how fish and olive oils ameliorate dysbiosis and depressive-like symptoms. Results suggest that fish oil, but not olive oil, improves depressive-like behavior and GM homeostasis in rats under chronic mild stress. In addition, Lai et al. (88) have evaluated how fish oil ameliorates the effects of gut dysbiosis in neuropsychiatric behaviors in rats under chronic sleep deprivation, accordingly with a reduction of proinflammatory markers in serum.

Another study has been performed to investigate the effects of fish and krill oils as supplements on a high-fat diet to reduce weight in mice (85). Results showed that dietary fish and krill oils were effective in the modulation of GM and the reduction of weight, liver index, and total cholesterol in high-fat diet-induced obesity mice. Lu et al. (83) also evaluated the role of different doses of dietary krill oil in modulating microbial communities at different gut locations (ileum and colon). Robertson et al. (84) investigated the effect of dietary marine lipids on pregnant mice and their male offspring. The study’s findings indicate that the addition of marine lipids from microalgae led to slight changes in the behavior of the male offspring and showed a strong ability to modulate cognitive function. The study found that the F/B ratio increased, indicating a shift in the overall composition of the GM. Additionally, the study found an increase in the abundance of the genera *Bifidobacterium* (Actinobacteria phyla) and *Lactobacillus* (Firmicutes phyla) (84). The effect of lipids from the microalgae Spirulina, as a dietary supplement, was investigated in rats fed a high-fat diet (86). The study found that this dietary intervention led to a reduction in hepatic lipid accumulation and steatosis, which are markers of non-alcoholic fatty liver disease (NAFLD). Additionally, the study found a decrease in the relative abundance of three bacterial genera *Turicibacter*, *Clostridium*_XIVa, and *Romboutsia,* that were positively associated with lipid metabolism.

Several studies (Table 1) have evaluated how a diet rich in marine n-3 PUFA leads to an increase in the presence of lactic acid bacteria and *Bifidobacterium* spp. in the human gut while saturated fatty acids disrupt the homeostasis of the GM components, promoting inflammation genera such as Bilophila or Bacteroides (93, 116). A high monounsaturated fatty acids diet during pregnancy can impact GM richness and diversity and potentially have negative effects, such as a relative increase of *Salmonella* spp. in feces. This highlights the importance of considering dietary fat quality and its effects on GM when designing interventions for pregnant women (117). In addition, Balfego et al. (92) studied how a diet rich in sardine affects type-2 diabetes, and the ratio of Phytoplankton/Bacteroidetes and TNFα levels were reduced after administration. Another study showed that enteral supplementation of a fish and safflower blend oil in premature infants with enterostomy led to greater bacterial diversity and a decrease in the abundance of some pathogenic bacteria, such as *Streptococcus, Clostridium, Escherichia, Pantoea, Serratia*, and *Citrobacter* (96). Noriega and colleagues (93) conducted a study in 2016 to analyze the effect of the supplementation of marine lipids for 2 weeks on the GM of a 45-year-old man. Results showed that fish lipid supplementation enhanced the abundance of the Firmicutes phylum while reducing the abundance of Bacteroidetes and Actinobacteria. Additionally, the *Faecalibacterium* genus was reduced, and the *Blautia, Roseburia, Coprococcus, Ruminococcus*, and *Subdoligranulum* genera abundance was increased; some of these genera are linked to the synthesis of the SCFA butyrate (93). Overall, this study is new evidence that marine lipids may have some effect on the composition of human GM, particularly on the content of specific beneficial bacterial taxa.

Nielsen and colleagues (94) investigated the implication of n-3 PUFAs in infant microbiota. Infants were fed with cow’s milk or infant formula w/o fish oil from 9 to 12th months of age. The results showed that fish oil supplementation in cow’s milk groups developed a different GM composition than in the infant formula groups. The same research group performed a similar study to evaluate microbiota composition in nine-month-old infants fed daily with fish oil or sunflower oil for 9 months (95). Results showed that fish oil could beneficially modify microbiota in comparison to sunflower oil; however, these changes were only visualized in infants who stopped breastfeeding before supplementations, suggesting that breastfeeding is also involved in microbiota composition. The authors suggested that cessation of breastfeeding allows the introduction of new bacteria to the infant gut microbiota, and thus supplementation with fish oil may have a greater impact on gut microbial composition in non-breastfed infants. In another study, in elderly population w/o HIV was administered fish oil supplements for 12 weeks. Gut barrier function and inflammatory factors related to the tract were evaluated, and the results showed fish oil supplements were able to reduce inflammation and intestinal permeability in this elderly population (118).

Therefore, in general, marine lipids have demonstrated capacity to modulate GM and improve gut dysbiosis by increasing the abundance and diversity of beneficial bacteria in animal models and in human studies, although more clinical studies are required to evaluate how factors such as the protocol for supplementation (time, doses), marine lipid compositions (ratio EPA/DHA), age (children vs. adult/elderly people), or even if infants are breastfed or formula fed, are modulating effect of marine lipids on the GM.

### 4.2 Marine lipids and food allergy

Several investigations support that marine lipids have the potential for anti-allergic activity by decreasing allergic symptoms. An enhancement in n-6/n-3 PUFA ratio cause disruption in the homeostasis of Th1/Th2; however, dietary marine n-3 LC-PUFAs are related to the reduction of Th2 and Th1 reactions, Treg cells frequency increases and IgE levels are decreased (119, 120).

Several animal models have been applied to investigate the effect of marine lipids (fish oils, EPA, DHA, or mixtures) on the development of FAs and related inflammatory processes. A study conducted on mice investigated the effects of feeding mice with EPA, DHA, or EPA + DHA, compared to corn oil (121). Results found that feeding mice with fish oil reduced the activation and differentiation of T cells in the spleen into pro-inflammatory Th17 cells compared to corn oil. This research also found that the expression of the pro-inflammatory cytokine IL-17 and the transcription factor RORγt were reduced without affecting Treg cell polarization in mice fed fish oil. Globally, these results show that marine lipids have a direct impact on the development of Th17 cells, which can be considered an anti-inflammatory mechanism via the suppression of this inflammatory T-cell subset (121). In addition, other studies have reported that fish oil reduces signs of FA in ovalbumin-sensitized mice (122) and prevents allergy sensitization to cow’s milk protein in mice (123). In this last investigation, the anti-allergy effect of marine lipids to cow’s milk allergy was related to an improvement of local intestinal and systemic Treg and a reduction of acute allergic indicators (123). Differences in the effect of EPA and DHA on FA have also been described. In particular, the supplementation of a DHA-rich fish oil was found to be more effective in suppressing allergic symptoms in mice allergic to whey and peanut compared to an EPA-rich fish oil (124).

Several clinical studies have been performed to evaluate the effects of marine lipids on allergies (Table 2). Results of multiple prospective studies found that lactating mothers who were fed diets rich in fish oils, such as tuna, salmon, or sardines, tend to have a higher EPA concentration in their breast milk. Furthermore, infants whose mothers consume these foods may have a lower risk of developing allergic diseases (133). Ellul et al. (134) found that the supplementation of fish oil in antenatal stages was related to higher levels of DHA and other n-3-related metabolites in infants at 1 year of age. This finding suggests that the intake of marine lipids during pregnancy may have benefits for infant health and development. On the contrary, higher levels of n-6 PUFAs were associated with an increased risk of developing FA (134). However, other studies that evaluated maternal supplementation with fish oil have found that offspring protection against food allergen sensitization was not related to maternal fish oil supplementation, although sensitization was lower than in the control group (127, 129). Similarly, another study performed on 657 infants breastfed from mothers administrated with tuna oil or soy oil supplements did not report a significant effect of fish oil supplementation on the incidence of FA (126).

**Table 2:** Summarized clinical studies investigating how the marine lipids administration and complement influence allergies*.

Diet	Sampling	Main outcomes	Reference
Daily administration of fish oil capsules (3.7 g of n-3 PUFAs, 56.0% of DHA, and 27.7% of EPA); or capsules of olive oil (66.6% n-9 oleic acid and <1% n-3 PUFAs), from 20 weeks gestation until delivery	40 atopic pregnant women	Offspring cytokine responses to all allergens decrease with fish oil administrations	Dunstan et al. (125)
Mothers were administrated with tuna oil (high-DHA diet, ∼1% total fatty acids) or soy oil (standard-DHA, ∼0.3% total fatty acids).	657 mothers with breastfed preterm infants born before 33 weeks gestation	There were no effects reported	Manley et al. (126)
Mothers were daily administrated with fish oil capsules (900 mg of n-3 PUFAs) or vegetable-origin oil capsules from 21 weeks gestation until delivery.	706 pregnant women with prenatal offspring at high risk of allergic disease development	n-3 PUFAs did not protect offspring against food allergen sensitization; lower atopic eczema and egg sensitization	Palmer et al. (127)
Daily administration of fish oil supplements (280 mg DHA and 110 mg EPA) or olive oil supplements from birth to 6 months of age.	420 infants at high atopic risk	There were no effects reported in the prevention of childhood allergic disease	D’Vaz et al. (128)
Daily administration of fish oil capsules (900 mg of n-3) or matched vegetable oil capsules from 21 weeks gestation until delivery	706 mothers with a fetus at high risk of development of allergic disease	There were no effects reported on sensitization or allergic disease symptoms.	Best et al. (129)
Administration of fish oil capsules (800 mg/ DHA and 100 mg/EPA) or vegetable oil capsules.	706 Six-year-old children with a family history of allergic disease	Prenatal n-3 PUFAs administration did not reduce IgE-associated allergy at 6 years of age. Decrease of children sensitized to house dust mite *Dermatophagoides farinae*.	Best et al. (130)
Fish oil, olive oil, or no oil in the ratio 2:1:1.	533 women during the third trimester of pregnancy	Asthma and allergic rhinitis medication prescribed was significantly reduced in the fish oil	Hansen et al. (131)
Mediterranean diet supplemented with two meals of 150 g of cooked fatty fish weekly for 6 months.	Children (aged 5–12 years) with mild asthma	Airway inflammation in childhood asthma was decreased with the supplementation of the Mediterranean diet.	Papamichael et al. (132)

*Studies mentioned in the manuscript with information about marine lipids supplementations obtained by questionnaires or similar were not added to this table.

Aldámiz-Echevarría et al. (135) evaluated the content of fatty acids in the plasma of children with multiple FAs, reporting significantly lower levels for the n-3 EPA and DHA compared to healthy children. This deficiency in n-3 EPA and DHA can be more dramatic in children with fish allergy (136), depending on how many fish species this individual is allergic to (137) since fish consumption is a major source of dietary EPA and DHA. Dunstan et al. (125) have reported in infants sensitized to hen’s egg through the skin, that allergy can be reduced by about one-half in infants of mothers who were previously administrated fish oil (3.7 g n-3 PUFAs per day) (125). D’Vaz et al. (128) found that daily fish oil supplementation, which contained 280 mg of DHA and 110 mg of EPA, improved the n-3 PUFA status of infants at high atopic risk. However, the study also found that fish oil administration did not protect against the occurrence of allergic outcomes, including sensitization, eczema, asthma, or FA, in infants at high atopic risk (128).

Although there is evidence suggesting that consuming fish during infancy and childhood may reduce the risk of developing allergic diseases, results from epidemiological studies are not entirely consistent (138). Different meta-studies have analyzed the effects of n-3 PUFA content of fish oil supplementation in pregnancy, lactation, infancy, or childhood, and discrepant results have been obtained. Fish oil administration is related to modifications in cytokine levels in cord blood and peripheral blood. These modifications provide the reduction of Th2 cytokine production; however there is less evidence about the effects on the Th1 response (71, 128, 138–143). The latest meta-analyses involved in the evaluation of the potential supplements of PUFAs given to infants or pregnant or breastfeeding mothers did not reveal any evidence of preventing the progress of asthma, dermatitis, or FA in infants or childhood (130, 144, 145). These results should be due to the use of different durations for supplementation and intakes, variable sources of PUFAs, and different n-3 to n-6 ratios (145, 146). Moreover, available results of antenatal fish oil administration on allergic respiratory diseases have not yet provided conclusive results (130, 131). Moreover, adults with diets rich in fish are suggested to prevent the development of inflammatory processes, such as rheumatoid arthritis (147), and enhance pulmonary function in asthma (132).

In summary, studies in animal models showed that the intake of marine lipids is able to decrease the outcomes for several FAs, according with a reduction on the activation and differentiation of T cells into pro-inflammatory variants, as well as the expression of pro-inflammatory cytokines. However, different clinical studies have produced inconclusive results regarding the potential benefits of marine lipids on fatty acids.

## 5 Conclusion

The present investigation provides the most recent information available about the impact that dietary marine lipids, essentially the n-3 EPA and DHA fatty acids consumed as supplements or directly in seafood, may have on modulating FAs and their immunological outcomes. FA is a biological response of the immune system to specific food components (allergens) with an important inflammatory component. Marine lipids can be physiologically metabolized to the bioactive lipid metabolites oxylipins that can regulate inflammation and immunity. In general, EPA-and DHA-derived lipid mediators are less inflammatory than those mediators related to n-6 ARA, and even some of them may act to resolve inflammation, reporting, in general, an anti-inflammatory effect. Dietary marine can also modulate GM structure and composition with potential human health benefits, such as in the case of FAs. Moreover, the intake of marine lipids is found to be effective in reducing activation and differentiation of T cells into pro-inflammatory T cell variants and expression of pro-inflammatory cytokines. Thus, abundant studies in animal models report the beneficial effects of dietary marine lipids to reduce outcomes for several FAs, although different clinical studies were found to be inconclusive about the potential beneficial effect of marine lipids on FA. To our best knowledge, these discrepancies in the results could be explained by a different mechanism of action of marine lipids depending on the food allergen, the use of diverse protocols for supplementation (time, doses), and fish oils with different compositions in terms of the content of EPA, DHA and, n-3 to n-6 ratios. In conclusion, the current study highlights the potential of marine lipids, whether consumed as a supplement or through seafood, to improve food allergies by reducing inflammation and regulating the gut microbiome and immune system. However, further systematic clinical investigations are required to tailor these beneficial effects to specific food allergies and individual circumstances. In the future, successful clinical applications of marine lipids can be expected to enhance the management of food allergies.

## Author contributions

AGA: Conceptualization, Writing – original draft, Writing – review & editing. MC: Conceptualization, Writing – original draft, Writing – review & editing. MP: Conceptualization, Writing – original draft, Writing – review & editing.
